# Understanding metabolic responses to forearm arterial occlusion measured with two-channel broadband near-infrared spectroscopy

**DOI:** 10.1117/1.JBO.29.11.117001

**Published:** 2024-11-29

**Authors:** Fiza Saeed, Caroline Carter, John Kolade, Robert Matthew Brothers, Hanli Liu

**Affiliations:** a University of Texas at Arlington, Department of Bioengineering, Arlington, Texas, United States; b University of Texas at Arlington, Department of Kinesiology, Arlington, Texas, United States

**Keywords:** broadband near-infrared spectroscopy, arterial occlusion, cytochrome c oxidase, metabolic activity, oxygenated hemoglobin

## Abstract

**Significance:**

Broadband near-infrared spectroscopy (bbNIRS) is useful for the quantification of cerebral metabolism. However, its usefulness has not been explored for broad biomedical applications.

**Aim:**

We aimed to quantify the dynamic responses of oxidized cytochrome c oxidase (Δ[oxCCO]) within the mitochondria to arterial occlusion and the dynamic correlations between hemodynamic (Δ[HbO]) and Δ[oxCCO] responses during and after occlusion in forearm tissues.

**Approach:**

We recruited 14 healthy participants with two-channel bbNIRS measurements in response to a 5-min forearm arterial occlusion. The bbNIRS system consisted of one shared white-light source and two spectrometers. The modified Beer-Lambert law was applied to determine the occlusion-induced changes in Δ[oxCCO] and Δ[HbO] in the shallow- and deep-tissue layers.

**Results:**

During the 5-min occlusion, dynamic responses in hemodynamics exhibited the expected changes, but Δ[oxCCO] remained constant, as observed in the 1- and 3-cm channels. A linear correlation between Δ[HbO] and Δ[oxCCO] was observed only during the recovery phase, with a stronger correlation in deeper tissues. The observation of a constant Δ[oxCCO] during the cuff period was consistent with two previous reports. The interpretation of this observation is based on the literature that the oxygen metabolism of the skeletal muscle during arterial occlusion remains unchanged before all oxy-hemoglobin (and oxy-myoglobin) resources are completely depleted. Because a 5-min arterial occlusion is not adequate to exhaust all oxygen supply in the vascular bed of the forearm, the local oxygen supply to the muscle mitochondria maintains redox metabolism uninterrupted by occlusion.

**Conclusions:**

We provide a better understanding of the mitochondrial responses to forearm arterial occlusion and demonstrate the usefulness of bbNIRS.

## 1 Introduction

Noninvasive measurement of the redox state of cytochrome c oxidase (CCO) using near-infrared spectroscopy (NIRS) was first proposed in 1977 by Jöbsis in a seminal Science article using 700 to 900 nm NIR light.^1^ Since then, NIRS has become an established detection tool for quantifying the absolute values of oxy- and deoxy-hemoglobin concentrations (i.e., [HbO] and [HHb], respectively) and their respective changes (Δ[HbO] and Δ[HHb], respectively) in tissue vasculature.^2^^–^^5^ NIRS-derived diffuse optical tomography using two to three NIR wavelengths has been well developed for non-invasive imaging of brain functions in both animals and humans with or without neurological diseases for 30 years or more.^3^^,^^5^ However, because the total concentration of CCO in tissues is much lower than that of hemoglobin ([HbT] = [HbO] + [HHb]), it was debated^6^^–^^9^ whether NIRS with several discrete NIR wavelengths (such as four to eight wavelengths) would be adequate for accurate quantification of the redox state of [CCO] (i.e., Δ[oxCCO]=[oxiCCO]−[redCCO]). This debate ended in 2012 to 2014 when broadband NIRS (bbNIRS) was introduced by numerous papers^10^^–^^13^ for its ability to accurately quantify Δ[oxCCO] and was further confirmed by comparing the results of Δ[oxCCO] with P31 metabolite peak-area ratios during and after transient cerebral hypoxia-ischemia in piglets.^14^ Since then, Δ[oxCCO] has been investigated as a sensitive marker of cerebral metabolism,^13^^,^^15^^,^^16^ as reviewed in several papers indicating the benefits of using bbNIRS to detect cerebral function in both neonates^17^ and adults.^18^^,^^19^

In the tissue optics or NIRS field, arterial occlusion of the human arm has commonly served as an experimental protocol or a living tissue model for the purpose of examining the quality of NIRS-based instruments, with the expectation that temporal changes in Δ[HbT] will remain constant, while changes in Δ[HbO] and Δ[HHb] will gradually decrease and increase, respectively, during the occlusion period. Meanwhile, forearm arterial occlusion is a research and clinical protocol used in cardiovascular research to study blood vessel health or function by measuring flow-mediated dilation (FMD) using a non-invasive Doppler ultrasound technique (most commonly in the brachial artery) for evaluating endothelial function and detecting vessel dilation.^20^^,^^21^ FMD has been shown to be predictive of cardiovascular diseases, such as cardiac death, myocardial infarction, and stroke, as a traditional risk factor.^22^^,^^23^

In most of these studies, little or no information was provided on any change in mitochondrial function, such as Δ[oxCCO], during occlusion because of a lack of (i) interest in it or (ii) appropriate equipment (e.g., bbNIRS) to measure it. For example, during forearm arterial occlusion, traditional Doppler ultrasound cannot detect any signal because of cessation of blood flow. This limitation does not exist in bbNIRS-based measurements. Our recent investigation of the rapid responses of Δ[oxCCO] to forearm arterial occlusion, as measured by bbNIRS, gave rise to intriguing findings, revealing that a temporal pause or stop of the redox reaction of CCO. Thus, the focus of this study was to assess the dynamic changes in Δ[oxCCO] at different tissue depths, along with hemodynamic alterations in Δ[HbO] and Δ[HHb] during and after forearm arterial occlusion. In the following sections, we present the experimental design, setup, and results, followed by a discussion to interpret our observation and to highlight the technical benefits of bbNIRS.

## 2 Materials and Methods

### 2.1 Research Participants and Exclusion Criteria

Our study comprised self-identifying African American and non-Hispanic Caucasian individuals, who reported that both biological parents were of the same race, reflective of the diverse population within the Dallas-Fort Worth Metroplex, Texas, United States. A total of 14 healthy participants (22.1±4.1 years of age) were recruited from February 2023 to November 2023 through a combination of flyers, social media, and word-of-mouth referrals. The inclusion criteria were recreationally healthy participants, with an age range of 18 to 35 years and body mass index (BMI) of 18.5 and $29.9\;\mathrm{kg}/m^{2}$.

Individuals who were tobacco users and competitive athletes were excluded from the study. Prospective participants were required to be free from overt cardiovascular, metabolic, and neurological diseases while refraining from the use of vasoactive prescription medications or supplements. Participants with a history of various microvasculature diseases, such as Reynaud’s disease, cold-induced urticaria, and cryoglobulinemia, were also ineligible. Furthermore, individuals currently taking prescription medications and those with a BMI exceeding $30\;\mathrm{kg}/m^{2}$ were excluded from the study. Given the impact of smoking on peripheral vasculature, both current smokers and individuals who had engaged in regular smoking (>1 pack per 2 weeks) within the previous 2 years were not considered eligible for participation.

### 2.2 Experimental Setup and Protocol of Two-Channel bbNIRS

**Fig. 1:**
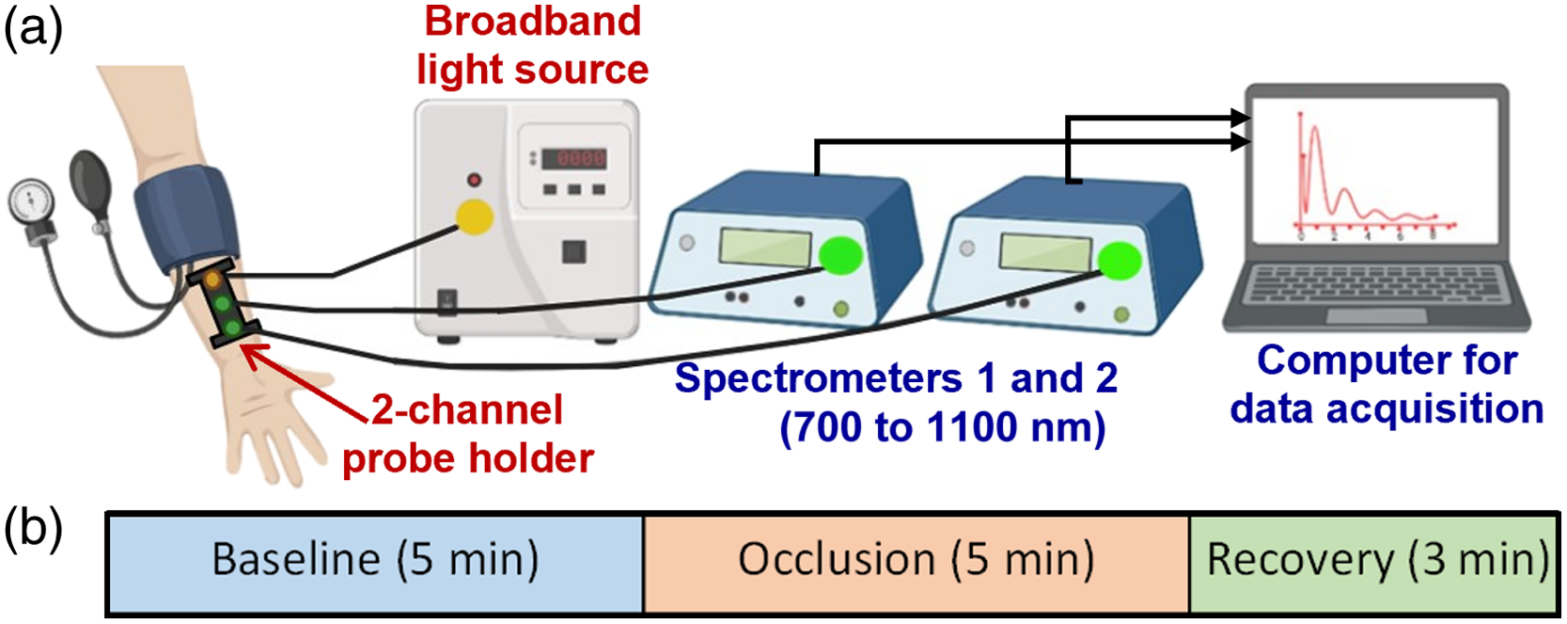
(a) It illustrates a schematic diagram showing the two-bbNIRS experiment setup; it consisted of a forearm cuff, a 3D-printed two-channel probe holder, two spectrometers, and a computer for data acquisition. (b) It presents the three-phase experimental protocol, including a 5-min baseline, 5-min arterial occlusion, and 3-min recovery period.

In this study, we utilized two-channel bbNIRS (2-bbNIRS), as shown in Fig. 1(a), comprising a white light source (OSL2IR, Thorlabs, Inc., Newton, NJ, United States) and two high-sensitivity broadband spectrometers (QEPRO, Ocean Optics Inc., Orlando, FL, United States) as light detectors.^24^^–^^26^ In one channel, the source and detector (S-D) separation was 1 cm to interrogate the signals within the shallow region (3 to 4 mm below the arm skin) of the forearm while the other channel had an S-D separation of 3 cm, enabling probing of dynamic changes in signals 1 to 1.5 cm in depth below the forearm skin. An integration time of 1.5 s (i.e., a sampling rate of 0.67 Hz) was set for both spectrometers to balance the temporal resolution and adequate signal strength. The two spectrometers were connected to a laptop computer that controlled the data acquisition, displayed the results, and stored the data for offline processing. Calibration of the two spectrometers was performed using an ink-intralipid phantom, demonstrating identical spectral quantification from both channels.

During the experimental setup, each participant laid down and relaxed their right arm before an automatic arm cuff was placed on the forearm just past their elbow [Fig. 1(a)]. Doppler ultrasound images of the brachial artery on the upper arm were obtained (but not shown because it was not the scope/interest of this study). A 3D-printed soft probe holder was attached to the brachioradialis muscle below the cuff proximal to the wrist and strapped securely to hold the optical source and detector fiber bundles steadily. The experimental protocol included three phases: 5-min baseline and 5-min forearm occlusion at a pressure of 220 mmHg for arterial occlusion, followed by a 3-min recovery after cuff release [Fig. 1(b)]. A pneumatic cuff was connected to a rapid inflation device (Hokanson Model E20 Rapid Cuff Inflator; Bellvue, WA, United States), which facilitated a rapid increase in cuff pressure to 220 mmHg within a few seconds. This protocol has often been used to study FMD in different populations.^27^^–^^29^ During the three phases of the experiment, the two-channel bbNIRS recorded continuously the time series over a 13-min period.

### 2.3 Data Processing and Calculations of Both Hemodynamic and Metabolic Parameters

Methods to quantify Δ[HbO], Δ[HHb], and Δ[oxCCO] have been developed and reported^11^^–^^13^ including our own methodology.^24^^,^^25^^,^^30^^,^^31^ A brief review is provided below for general readers who wish to understand the theoretical foundation and processing methods in depth. Figure 6 in Appendix A illustrates graphically the processing steps, which are described below.

Step 1: A broadband near-infrared spectroscopy (bbNIRS) system provides measurements of optical spectra at different times (t), as expressed I(t,λ).
Step 2: A relative optical density spectrum, ΔOD(t,λ), can be defined and calculated at each wavelength λ as follows:^30^^,^^31^
  $$\Delta \mathrm{OD}(t,\lambda )=\log_{10}[\frac{I_{0}(t=0,\lambda )}{I(t,\lambda )}],$$ (1)
  where $I_{0}(t=0,\lambda )$ can be the baseline spectrum at time t=0 or an average of several initial baseline spectral readings, and I(t,λ) represent time-varying spectra acquired at each time point throughout the entire experiment.
Step 3: The estimations of Δ[HbO] and Δ[oxCCO] from raw spectral data taken with bbNIRS throughout the experiment were based on modified Beer-Lambert’s law, which offers a quantitative relationship of ΔOD(λ) on Δ[HbO], Δ[HHb], and Δ[oxCCO] at each wavelength, λ, at each time point, with a wavelength-dependent path-length, L(λ). Based on the modified Beer-Lambert law,^4^^,^^32^ ΔOD(λ)/L(λ) can be expressed as a sum of optical absorbance contributed by Δ[HbO], Δ[HHb], and Δ[oxCCO] components, as given below
  $$[\begin{array}{l} \frac{\Delta \mathrm{OD}(\lambda_{1})}{L(\lambda_{1})}\\ \frac{\Delta \mathrm{OD}(\lambda_{2})}{L(\lambda_{2})}\\ \frac{\Delta \mathrm{OD}(\lambda_{3})}{L(\lambda_{3})}\\ \ldots \\ \frac{\Delta \mathrm{OD}(\lambda_{n})}{L(\lambda_{n})} \end{array}]=\Delta [\mathrm{HbO}]*[\begin{array}{l} \varepsilon_{\mathrm{HbO}}(\lambda_{1})\\ \varepsilon_{\mathrm{HbO}}(\lambda_{2})\\ \varepsilon_{\mathrm{HbO}}(\lambda_{3})\\ \ldots \\ \varepsilon_{\mathrm{HbO}}(\lambda_{n}) \end{array}]+\Delta [\mathrm{HHb}]*[\begin{array}{l} \varepsilon_{\mathrm{HHb}}(\lambda_{1})\\ \varepsilon_{\mathrm{HHb}}(\lambda_{2})\\ \varepsilon_{\mathrm{HHb}}(\lambda_{3})\\ \ldots \\ \varepsilon_{\mathrm{HHb}}(\lambda_{n}) \end{array}]+\Delta [\mathrm{CCO}]*[\begin{array}{l} \varepsilon_{\mathrm{CCO}}(\lambda_{1})\\ \varepsilon_{\mathrm{CCO}}(\lambda_{2})\\ \varepsilon_{\mathrm{CCO}}(\lambda_{3})\\ \ldots \\ \varepsilon_{\mathrm{CCO}}(\lambda_{n}) \end{array}],$$ (2)
  where $\varepsilon_{\mathrm{HbO}}(\lambda )$ and $\varepsilon_{\mathrm{HHb}}(\lambda )$ represent the extinction coefficients at each wavelength of HbO and HHb, respectively, and $\varepsilon_{\mathrm{CCO}}(\lambda )$ denotes the difference in extinction coefficient between oxidized and reduced CCO, which can be found in Refs. 11 and 17. All extinction coefficients given in Refs. 11 and 17 were defined using the base-10 logarithm, as shown in Eq. (1). The corresponding extinction coefficients using the natural logarithm can be found in Ref. 33 with a factor of 2.3.
  Furthermore, according to the modified Beer-Lambert Law,^4^^,^^32^ L(λ) can be expressed as
  $$[\begin{array}{l} L(\lambda_{1})\\ L(\lambda_{2})\\ L(\lambda_{3})\\ \ldots \\ L(\lambda_{n}) \end{array}]=r*[\begin{array}{l} \mathrm{DPF}(\lambda_{1})\\ \mathrm{DPF}(\lambda_{2})\\ \mathrm{DPF}(\lambda_{3})\\ \ldots \\ \mathrm{DPF}(\lambda_{n}) \end{array}],$$ (3)
  where r is a constant that denotes the source-detector distance. In this study, we used S-D separations of 1 and 3 cm, so r=1 and 3 cm, respectively. The wavelength dependence of L(λ) is caused by a wavelength-dependent differential pathlength factor [DPF(λ)].
Step 4: By substituting Eq. (3) into Eq. (2) for multiple wavelengths, the estimation of Δ[HbO], Δ[HHb], and Δ[oxCCO] can be expressed as follows:
  $$[\begin{array}{l} \Delta [\mathrm{HbO}]\\ \Delta [\mathrm{HHb}]\\ \Delta [\mathrm{CCO}] \end{array}]=\frac{1}{r}*{\left[\begin{array}{ccc} \varepsilon_{\mathrm{HbO}}(\lambda_{1}) & \varepsilon_{\mathrm{HHb}}(\lambda_{1}) & \varepsilon_{\mathrm{CCO}}(\lambda_{1})\\ \varepsilon_{\mathrm{HbO}}(\lambda_{2}) & \varepsilon_{\mathrm{HHb}}(\lambda_{2}) & \varepsilon_{\mathrm{CCO}}(\lambda_{2})\\ \ldots & & \\ \varepsilon_{\mathrm{HbO}}(\lambda_{n}) & \varepsilon_{\mathrm{HHb}}(\lambda_{n}) & \varepsilon_{\mathrm{CCO}}(\lambda_{n}) \end{array}\right]}^{-1}[\begin{array}{l} \frac{\Delta \mathrm{OD}(\lambda_{1})}{\mathrm{DPF}(\lambda_{1})}\\ \frac{\Delta \mathrm{OD}(\lambda_{2})}{\mathrm{DPF}(\lambda_{2})}\\ \ldots \\ \frac{\Delta \mathrm{OD}(\lambda_{n})}{\mathrm{DPF}(\lambda_{n})} \end{array}].$$ (4)

To accurately solve Δ[HbO], Δ[HHb], and Δ[oxCCO] using Eq. (4), we needed to know DPF(λ) in the wavelength range of our measurements. In the literature, there are three major methods to determine the DPF using data-driven, model-fitting, or the two combined approaches. First, the data-driven method was based on time-resolved NIRS measurements of the human tissue under study in a group of healthy subjects, followed by calculations of the mean time-of-flight (⟨t⟩) of light traveling in the tissue at the group level.^17^^,^^34^ The group-averaged time-of-flight multiplied by the speed of light within the tissue results in the total optical pathlength, ⟨L⟩, where ⟨L⟩=DPF × source-detector separation. This approach has been generalized for n wavelengths (termed UCLn) and is used for bbNIRS measurements without additional time- or frequency-domain measurements. Second, in the model-based approach, “the DPF” can be quantified using diffusion theory if both the light absorption and reduced scattering coefficient of the tissue are determined by time- or frequency-domain measurements.^35^ Finally, a hybrid method of data-driven and model-fitting combination was also reported by combining the UCLn, diffusion theory fitting, and fitting of the 2nd derivative of the measured reference spectrum to the second derivative of the water absorption spectra.^18^ Comparisons of the derived Δ[oxCCO] and Δ[HbO] values using three different DPF calculation algorithms are desirable but not the scope of this study.

In this study, we took the diffusion-theory approach and quantified the DPF with the semi-infinite boundary geometry,^36^ as expressed below

$$\mathrm{DPF}(\lambda )=\frac{\sqrt{3\mu_{s}^{\prime }(\lambda )}}{2\sqrt{\mu_{a}(\lambda )}}*\frac{r\sqrt{3\mu_{a}(\lambda )\mu_{s}^{\prime }(\lambda )}}{r\sqrt{3\mu_{a}(\lambda )\mu_{s}^{\prime }(\lambda )}+1},$$ (5)

where $\mu_{a}(\lambda )$ and $\mu_{s}^{\prime }(\lambda )$ are the estimated absorption and reduced scattering coefficients across the wavelength range of interest. Also, we assumed DPF(λ) values to be time-invariant because of stable optical properties of the human arm. The DPF given in Eq. (5) does not require any definition using the base-10 or base-e logarithm because the $\mu_{a}(\lambda )$ and $\mu_{s}^{\prime }(\lambda )$ values of the tissue are already determined within the diffusion theory calculations.

Values of $\mu_{a}(\lambda )$ and $\mu_{s}^{\prime }(\lambda )$ were measured using a tissue oximeter (OxiplexTS, ISS) that operates in the frequency domain. This device provides readings of $\mu_{a}$ and $\mu_{s}^{\prime }$ values at 750 and 830 nm, as well as absolute concentrations of [HbO] and [HHb].^36^ However, to obtain $\mu_{s}^{\prime }(\lambda )$ values across the entire range of wavelengths from 780 to 900 nm, we used Mie theory to interpolate and extrapolate the two measured $\mu_{s}^{\prime }$ values at 750 and 830 nm. Mie theory is typically represented by $k\lambda^{-b}$, where k and b were determined by fitting this equation to both $\mu_{s}^{\prime }$ values at 750 and 830 nm.^37^ In addition, absorption coefficients in the same wavelength range (780 to 900 nm) were estimated based on [HbO] and [HHb] measured by the same tissue oximeter.^30^

After combining the measured ΔOD(λ) values across the measurement period (13 min) and the group-averaged $\mu_{a}(\lambda )$ and $\mu_{s}^{\prime }(\lambda )$ values of the human forearm derived from the frequency-domain NIRS measurements,^30^ we were able to solve Eq. (4) at each measurement time point using MATLAB, achieving temporal series of Δ[HbO], Δ[HHb], and Δ[oxCCO] under respective experimental conditions. Specifically, our calculations covered the spectral range of 780 to 900 nm with a total of 121 wavelengths.

Group-level averages of Δ[HbO], Δ[HHb], and Δ[oxCCO] were obtained for each of the three phases (i.e., 5-min baseline, 5-min during forearm cuff, and 3-min recovery). Standard errors of the mean were used to plot error bars in each respective figure.

## 3 Results

### 3.1 Hemodynamic and Metabolic Responses to 5-min Forearm Arterial Occlusion

**Fig. 2:**
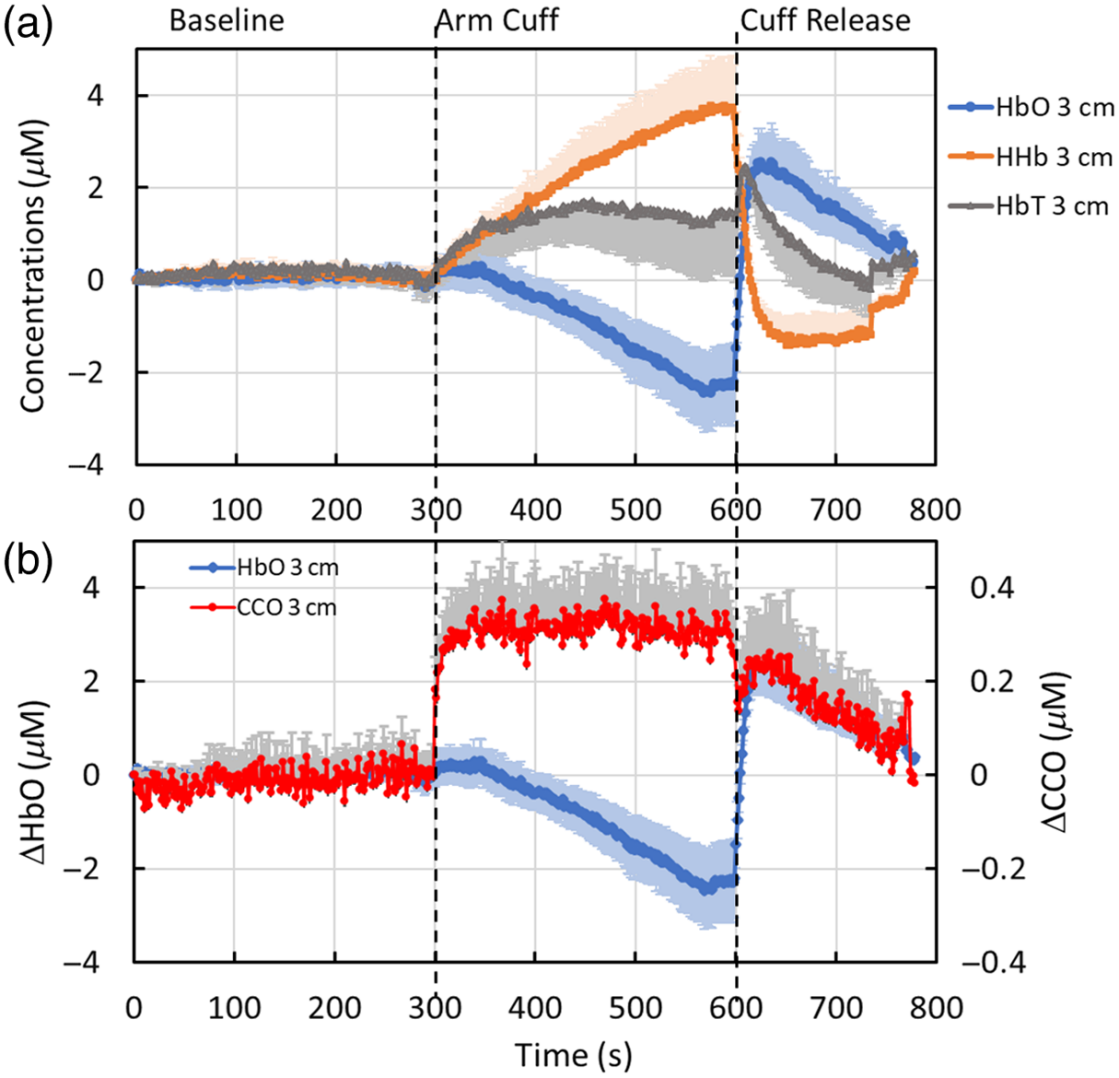
(a) Dynamic changes in Δ[HbO] (blue), Δ[HHb] (orange), and Δ[HbT] (gray) during the 5-min baseline, 5-min forearm arterial cuff, and 3-min cuff release (n=14). The x-axis represents time in seconds, whereas the y-axis depicts the changes in the respective concentrations in μM. (b) Dynamic changes in Δ[oxCCO] (red) are plotted and compared with changes in Δ[HbO] (blue). The y-axis for Δ[oxCCO] is labeled on the right side of the figure, which is an order of magnitude smaller than that for Δ[HbO] on the left side of the figure. In both panels, the error bars represent the standard error of the mean for the respective concentrations. For Δ[HHb] (orange), Δ[HbT] (gray), and Δ[oxCCO] (red), only half of the error bars (either above or below the curves) are shown to avoid excessive bar overlap.

Figure 2(a) shows the group-level temporal series of Δ[HbO], Δ[HHb], and Δ[HbT] during the three continuous phases obtained from the 3-cm channel. During the 5-min forearm arterial occlusion, the results clearly show a gradual decrease in Δ[HbO] (blue curve), a corresponding increase in Δ[HHb] (orange curve), and a relatively constant Δ[HbT] (gray curve) after a small initial increase. Following cuff release, all three concentrations rapidly returned to their baselines with overshoots during the 3-min recovery period. The three observed dynamic patterns were well expected and aligned perfectly with the underlying physiology, as reported in the literature.

However, Fig. 2(b) presents an interesting dynamic pattern of Δ[oxCCO] (red curve). During the 5-min forearm arterial occlusion, Δ[oxCCO] was not reduced in the same manner as Δ[HbO]; instead, Δ[oxCCO] remained constant after an initial rapid jump. After the cuff was released, Δ[oxCCO] returned to baseline with a dynamic course similar to that of Δ[HbO] during the 3-min recovery period. Since the y-axis for Δ[oxCCO] is labeled a factor of 10 smaller than that for Δ[HbO], the cuff-induced changes in Δ[oxCCO] were ∼10 times smaller than that of Δ[HbO], which matches the ratio between the concentrations of these two chromophores in living tissues, such as the human forearm.

### 3.2 Relationship Between Δ[HbO] and Δ[oxCCO] Measured with the 3-cm Channel

**Fig. 3:**
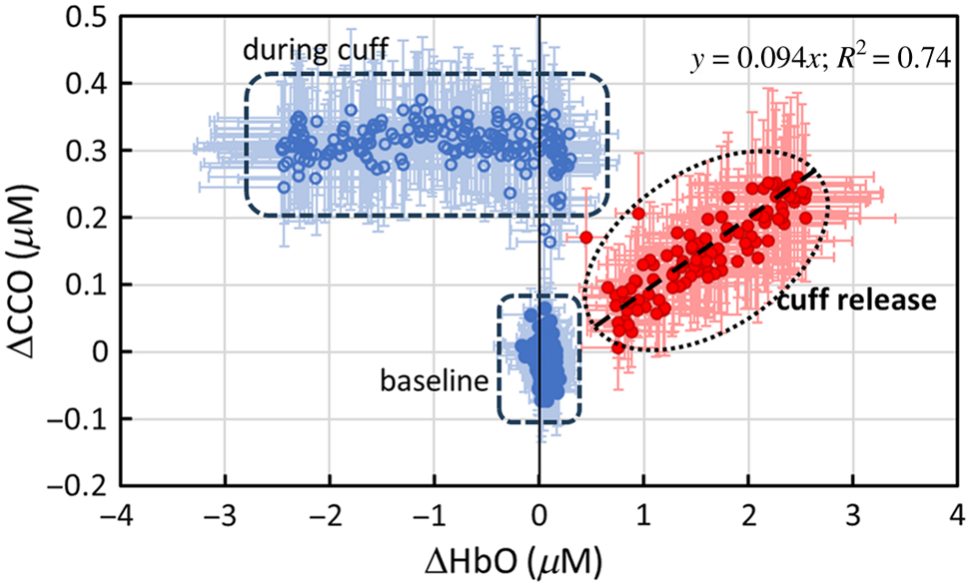
Relationships between Δ[HbO] and Δ[oxCCO] measured with the 3-cm channel during the three dynamic phases, namely, 5-min baseline (blue solid circles), 5-min arm cuff (blue open circles), and 3-min cuff release (red circles). A linear fit for the data after cuff release was obtained and is shown by the black dashed line with the fitting equation of y=0.094x and $R^{2}=0.74$. The error bars represent the standard error of the mean for both Δ[HbO] and Δ[oxCCO].

Given the observed but unexpected dynamic course of Δ[oxCCO], it was necessary to further investigate the relationship between Δ[oxCCO] and Δ[HbO]. Figure 3 plots Δ[oxCCO] versus Δ[HbO] for the 5-min baseline, 5-min arterial occlusion, and 3-min cuff release (with the 3-cm S-D separation). This indicates that three clusters were formed corresponding to the three dynamic phases. The changes in the two chromophores did not show any correlation during the baseline and cuff periods (blue markers), while Δ[oxCCO] and Δ[HbO] showed a strong linear correlation during the recovery phase, with a slope of ∼0.1 and an $R^{2}$ of 0.74 (or an R value of 0.86). These results imply that the coupling between the changes in HbO and oxCCO during the recovery phase was high, whereas these two quantities were completely uncoupled during arterial occlusion.

### 3.3 Dynamic Relationships of Each of Δ[HbO], Δ[HHb], and Δ[oxCCO] Between Shallow and Deeper Forearm Tissues Measured Using 1- and 3-cm Channels

**Fig. 4:**
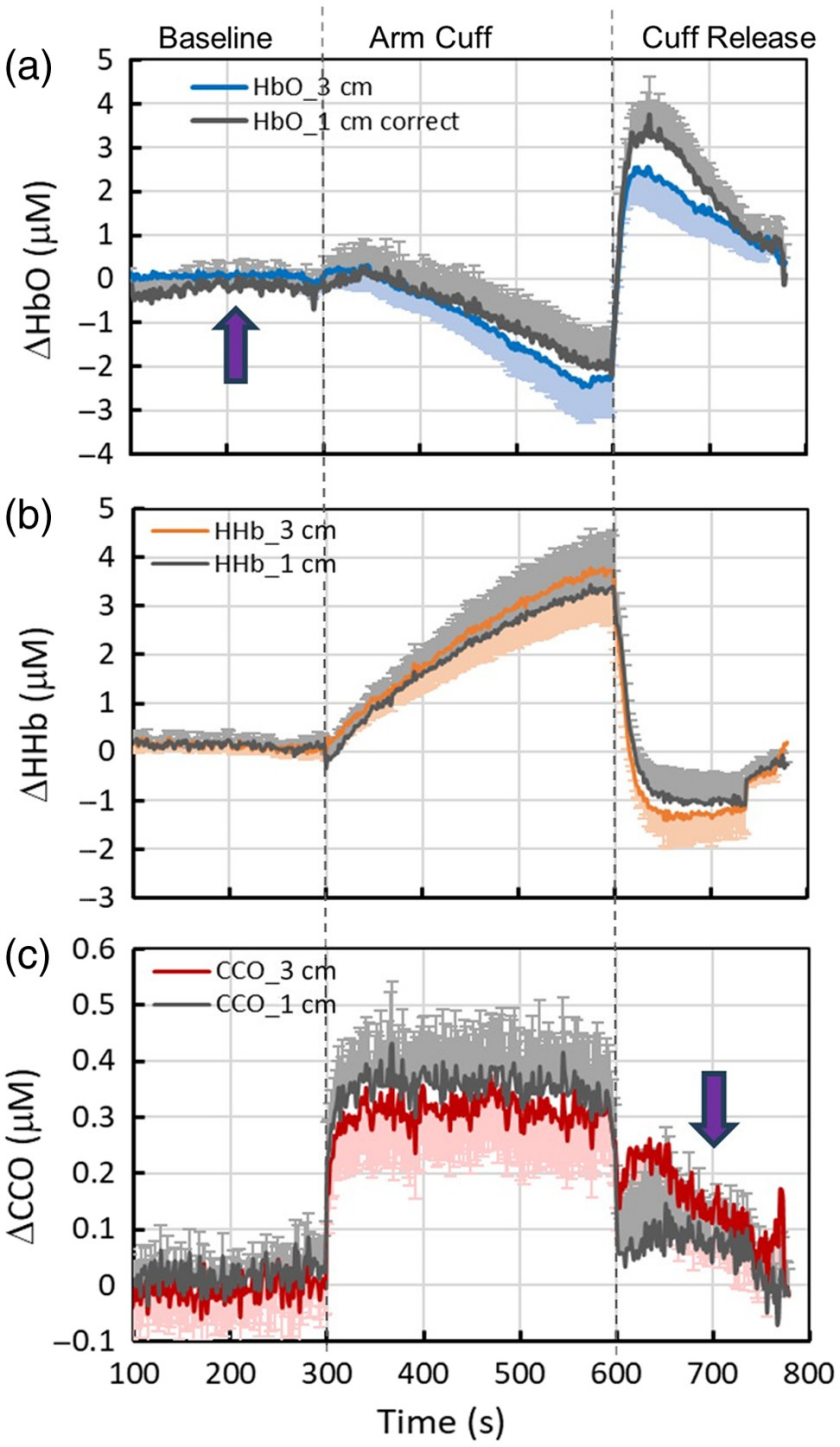
Dynamic courses of (a) Δ[HbO], (b) Δ[HHb], and (c) Δ[oxCCO] recorded by 1- and 3-cm channels, respectively, throughout the three measurement phases. In all three panels, the error bars represent the standard error of the mean for the respective concentrations, with only half of the error bars (either above or below the curves) shown to avoid excessive bar overlap. The two purple arrows indicate a low correlation between the two channels, namely, during the baseline for Δ[HbO] ($R^{2}=0.3$) and during the recovery for Δ[oxCCO] ($R^{2}=0.5$). See Fig. 7 in Appendix B for detailed information about the low correlations.

Because our measurements were taken using both 1- and 3-cm S-D channels, we investigated and compared the dynamic traces/courses of Δ[HbO], Δ[HHb], and Δ[oxCCO] recorded by both channels. Figures 4(a)–4(c) present comparisons for each of the three chromophores. Note that the starting time in all three panels was 100 s after an initial drift in the first 100 s for the 1-cm channel was removed or corrected. It is noted that both the 1- and 3-cm channels resulted in similar changes in magnitude and time course for both Δ[HHb] [Fig. 4(b)] and Δ[oxCCO] [Fig. 4(c)] but gave rise to different dynamic magnitudes in Δ[HbO] during the second half of the cuff period and recovery [Fig. 4(a)]. To specifically reveal the dynamic relationships of each chromophore taken with 1- and 3-cm channels, Fig. 7 in Appendix B shows linear correlations between each pair of channels for each chromophore during each of the three dynamic phases. The correlations between the two channels were high ($R^{2}>0.7$) in most cases (seven out of nine cases), except in two situations: (i) during the baseline for Δ[HbO] [Fig. 7(a); $R^{2}=0.3$] and (ii) during the recovery phase for Δ[oxCCO] [Fig. 7(i); $R^{2}=0.5$]. These two special cases are marked in Figs. 4(a) and 4(c) by purple arrows, respectively.

**Fig. 5:**
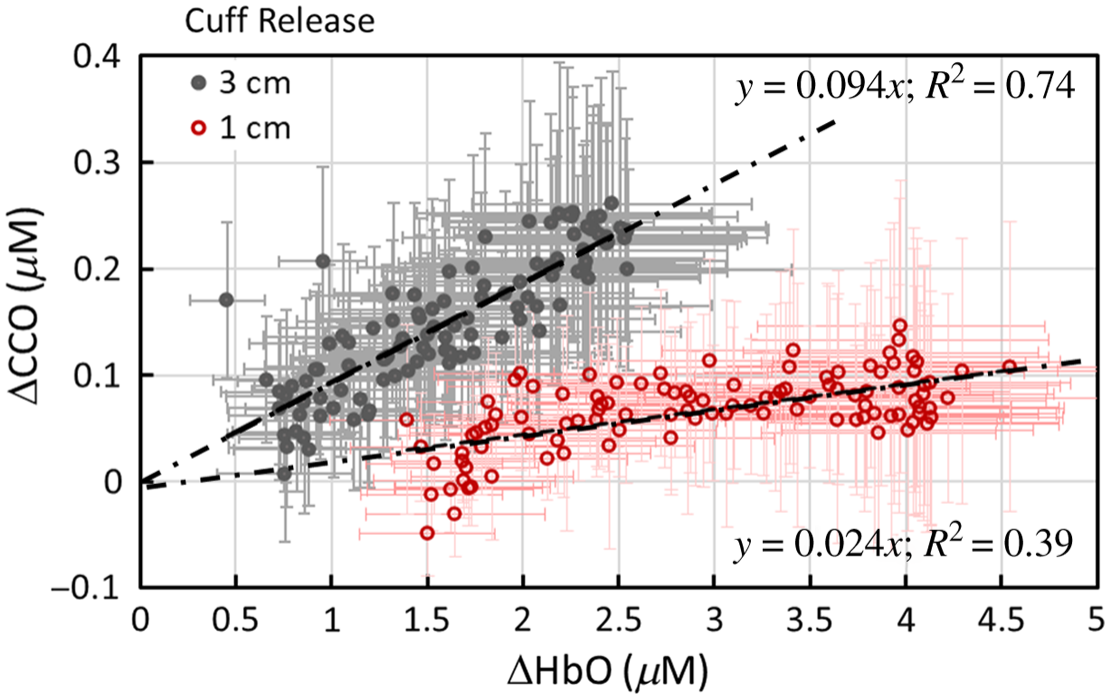
Correlations between Δ[HbO] and Δ[oxCCO] obtained from the 1-cm (red) and 3-cm (dark gray) channels. The error bars represent the standard error of the mean for the respective concentrations. Both sets of data were fitted with a linear regression with $R^{2}$ values of 0.39 and 0.74 for the 1- and 3-cm channels, respectively.

Given a relatively low correlation between the two channels of Δ[oxCCO] during the recovery phase, we plotted Δ[oxCCO] versus of Δ[HbO] from both channels, as shown in Fig. 5, with the data from the 3-cm channel replotted here (dark gray markers) for easy comparison. Given that the 1-cm channel is more sensitive to superficial layers of tissue (<5  mm), this figure reflects that the changes in Δ[oxCCO] and Δ[HbO] during the recovery phase were much less coupled or much weaker interacted in the shallow layer of the forearm tissue (as detected by the 1-cm channel) than that in the deeper region (>5  mm) of the tissue.

## 4 Discussion

### 4.1 Understanding Unchanged Δ[oxCCO] During the Forearm Arterial Occlusion

One key and intriguing observation of this study is the unchanged or constant time course of Δ[oxCCO] during the 5-min arterial occlusion on the forearm, as shown in Figs. 2(b) and 4(c). With a complete lack of blood and oxygen supplies by arterial occlusion, the oxy-hemoglobin concentration, Δ[HbO], within the forearm vasculature is expected to decrease gradually at either shallow or deep depths, as seen in Figs. 2(a) and 4(a). However, according to the literature,^38^^–^^40^ skeletal muscle oxygen metabolism during arterial occlusion should remain unchanged with respect to that prior to arterial occlusion, before all oxy-hemoglobin (and oxy-myoglobin) resources are completely depleted. Several published studies have shown that a 5-min arterial occlusion period is not adequate to exhaust all oxygen supply in the vascular bed of the forearm during arterial occlusion.^39^^,^^41^ Thus, the local oxygen supply to cellular mitochondria continues and maintains redox metabolism uninterrupted by occlusion. Accordingly, temporal changes in Δ[oxCCO] stayed idle or constant during the 5-min cuff period.

Our observation of a constant Δ[oxCCO] during forearm occlusion is consistent with numerous reports from previous publications. As reviewed in Ref. 19, Lange et al.^39^ presented a constant temporal profile of Δ[oxCCO] during a 5-min arterial occlusion using two independent quantification methods: the modified Beer-Lambert law and diffusion theory. In addition, Islam et al.^42^ reported a relatively small increase in Δ[oxCCO] in the initial cuff period (∼1  min), followed by a constant profile for the remaining time of 4-min venous occlusion. The small initial increase was not statistically tested for its significance, and the low temporal resolution limited the accuracy of the initial onset of Δ[oxCCO] increase. The overall agreement between our results and those of these two studies is that the responses of Δ[oxCCO] during the 4- or 5-min forearm occlusion remained constant and did not follow the dynamic patterns or behaviors of Δ[HbO]. Previous studies^39^^,^^42^ and a recent review^19^ supported that the unchanged Δ[oxCCO] during the cuff period indicates the minimization or absence of crosstalk between Δ[oxCCO] and Δ[HbO] by the quantification algorithm because the former does not mirror the latter signals. However, none of these studies explicitly provided and stated the underlying principles of this observation.

### 4.2 Physiological Meanings of 1-cm Versus 3-cm bbNIRS Measurements

It is well known that the measurement readings from 1-cm S-D separation of an NIRS or bbNIRS (bb/NIRS) device are more sensitive to superficial tissues within a few millimeters in depth, whereas a 3-cm channel of bb/NIRS can detect hemodynamic (and metabolic) signals in deeper regions of living tissues, at least 1 to 2 cm below the tissue skin or surface depending on different types of tissues. The current study reported several observations.

First, being a soft tissue with a limited depth on the human forearm, most of the dynamic courses for Δ[HbO], Δ[HHb], and Δ[oxCCO] with 1- and 3-cm channels were highly correlated before, during, and after the occlusion phases [Figs. 7(b)–7(h)]. This means that there is no significant depth-resolved or layer-resolved difference along the human forearm with respect to the hemodynamic and metabolic properties or heterogeneity detected by bb/NIRS in either resting/static or dynamic processes. However, there are two special cases, as shown in Figs. 7(a) and 7(i), as discussed further next.

Second, the weak correlation between the 1- and 3-cm channels for Δ[HbO] [Fig. 7(a); $R^{2}=0.3$] during the baseline is understandable and interpretable. Because the bb/NIRS is a relative measurement with respect to a selected time point (i.e., the first time point in this case), the time series of Δ[HbO] acquired from the two channels may include complex vasomotion-related components, covering different infraslow oscillation frequencies^24^^,^^26^ that can be heterogenous at different depths during the baseline phase. Consequently, the baseline Δ[HbO] time series originating from different depths may be less correlated, as observed in this study.

Third, the moderate correlation during the recovery phase for Δ[oxCCO] [Fig. 7(i); $R^{2}=0.5$] may be associated with the depth-resolved redox state of CCO in response to reperfusion. In other words, there must be a stronger coupling between mitochondrial/metabolic and hemodynamic activity in the deeper forearm tissue region, where a more concentration of mitochondria exists in the muscle cells than in the superficial layers (consisting of the skin and fatty tissues). A higher oxygen demand in the deeper tissue region (seen by the 3-cm channel) would dictate a greater blood supply and thus a tighter coupling with oxygenated blood. Thus, during the reperfusion phase, the dynamic courses of Δ[oxCCO] at different arm tissue depths were partially correlated [$R^{2}=0.5$; Fig. 7(i)], but not highly correlated, in contrast to Δ[HbO] and Δ[HHb] [$R^{2}>0.9$; Figs. 7(g) and 7(h)]. Indeed, Fig. 5 also supports this interpretation, showing that the correlation between Δ[oxCCO] and Δ[HbO] was significantly higher in the deeper region (measured by the 3-cm channel) than in the superficial layer seen by the 1-cm channel.

### 4.3 Demonstration of bbNIRS as a Useful Device to Quantify Tissue Metabolism

This study has two key benefits for the research field. First, it has been a concern for years that it is difficult to determine Δ[oxCCO] independently and accurately because of the possible crosstalk between Δ[HbO] and Δ[oxCCO] in the algorithm. In the limited number of studies that reported Δ[oxCCO], the signal changes in Δ[HbO] and Δ[oxCCO] always followed the same direction or trend (i.e., they either increased or decreased together).^17^ However, this study unambiguously showed that during the 5-min arterial occlusion, Δ[oxCCO] remained constant, while Δ[HbO] gradually decreased. This evidence proves that the bbNIRS algorithm enables the separation of two chromophores with different changing directions or trends. Second, this study selected an excellent example to demonstrate that bbNIRS is a very useful device for noninvasive measurement and quantification of the redox state of CCO, which may serve as a clinical marker or feature to investigate critical tissue metabolism, such as for a better understanding of remote ischemic pre/conditioning (RIPC/RIC) for future clinical applications. Third, this study is more specific for the investigation of reperfusion injury that occurs in many medical applications, such as stroke and cardiac arrest. Sudden reintroduction of oxygen during reperfusion can lead to a burst of reactive oxygen species (ROS) production, which can cause further damage to CCO and other components of the mitochondria, leading to ischemia-reperfusion injury. bbNIRS can be a convenient and non-invasive tool for monitoring the state of CCO and thus to predict or reveal potential damage of mitochondria by reperfusion.

### 4.4 Noninvasive Tool to Study Remote Ischemic Pre/Conditioning

RIC/RIPC is a process that applies a blood pressure cuff to a limb above the systolic pressure for short periods of time to protect against ischemia in other vascular areas. The RIC stimulus can be applied prior to ischemia (preconditioning), after the onset of ischemia (preconditioning), or at the time of reperfusion (postconditioning). Its physiological principle is that ischemic conditioning (such as arm occlusion) introduces or stimulates an endogenous protective mechanism that allows organisms to develop resistance to subsequent insults.^43^^–^^45^ The three major proposed mechanisms or pathways for transmitting protective signals from the organ or tissue to the heart and brain are (i) the neural pathway, (ii) the release of circulating humoral factor(s), and (iii) the activation of a systemic protective effect (such as an anti-apoptotic or anti-inflammatory response).^43^^–^^46^ Numerous publications over the past 10 to 12 years have reported that RIC/RIPC has shown potential benefits in stroke recovery and rehabilitation research.^47^ However, its use in this context is still an area of ongoing study, and its effectiveness and optimal protocols have not yet been fully established.^47^^,^^48^

The rapid arterial occlusion protocol used in this study was very similar to a portion or the initial part of the RIPC/RIC strategy.^43^^–^^46^ It was suggested by Saccaro et al., “Most of the pathways activated by RIC are believed to ultimately affect the mitochondria, preventing for example the formation of the mitochondrial permeability transition pore (MPTP)… This could reduce the generation of mitochondrial reactive oxygen.”.^45^ Accordingly, it may be helpful to perform non-invasive bbNIRS and quantify metabolic or mitochondrial responses to RIC/RIPC (e.g., during and after repeated arm arterial occlusion). Such measurements can be taken in multiple locations, including a remote site, such as on both arms or both legs, one being on the arm/leg with arterial occlusion and the other being remote. All of these prospective studies will enrich the scientific literature for a better understanding of RIC/RIPC.

### 4.5 Limitations of this Study and Future Work

This study had two major limitations. First, based on physiology and many observed arterial occlusion results using conventional two-wavelength NIRS, one would expect to observe a constant Δ[HbT] within several seconds after full arterial occlusion is reached. However, Fig. 2(a) of this study shows that the temporal change in Δ[HbT] continued to increase over the initial 50 to 60 s of cuff occlusion even though the cuff was fully inflated to 220 mmHg within 5 to 10 s. Similarly, a delayed time to reach an equilibrium of Δ[HbT] was also implied in Ref. 39 during the 1 to 1.5 min onset of arterial occlusion (when adding both Δ[HbO and Δ[HHb]), which were consistent between the results using two analysis methods. Reference 39 attributed the temporal delay in reaching a constant Δ[HbT] to the sequence of venous occlusion followed by arterial occlusion, causing an augmentation of Δ[HbO] and thus Δ[HbT] in the initial onset of cuff inflation. Unfortunately, the number of published papers investigating hemodynamic and redox activities of the human forearm in response to arterial occlusion is limited. For example, Ref. 42 used a 4-min venous occlusion protocol to record Δ[CCO] changes and thus is unable to provide the needed information. Thus, it is highly desirable to have more similar experiments to be conducted and investigated in the field for rigorous scientific confirmation and exploration.

Second, in this study, we observed a sudden increase in Δ[oxCCO] [Figs. 2(a) and 4(c)], which was not seen in Refs. 39 and 42. However, neither of these references used a broadband NIRS with 100+ wavelengths, so it is unclear whether our measured sudden rise in Δ[oxCCO] was caused by any artifact or a possible algorithm crosstalk between oxCCO and HbO. For the latter, we do not believe to be the source since all other temporal patterns of Δ[HbO], Δ[HHb], and Δ[oxCCO] are well expected or explained. Our speculation of this observation is as follows. When the local blood and oxygen supply to a tissue is occluded, the affected mitochondria undergo a series of acute changes or insults, including perhaps an opening of the MPTP and increased ROS production, some of which may lead to mitochondrial swelling. The latter indicates an increase in mitochondrial size. Based on a mathematical model of light-tissue interaction,^49^ a decrease in light scattering (denoted by $\mu_{s}^{\prime }(\lambda )$) occurs when mitochondrial size increases. A reduced $\mu_{s}^{\prime }(\lambda )$ leads to a reduction in DPF [see Eq. (5)] and, consequently, to an increase in Δ[oxCCO] [see Eq. (4)]. This hierarchical cascade in math could be, at least partially, the underlying physics/physiology for interpreting the sudden increase in Δ[oxCCO] at the initial arterial occlusion. Such speculation requires further investigation and confirmation by quantifying Δ[oxCCO] changes in response to forearm arterial occlusion with repeated experiments and a larger sample size of healthy participants.

## 5 Conclusions

In this study, we aimed to demonstrate a broader application of bbNIRS by focusing on the quantification of dynamic changes in Δ[oxCCO] at different tissue depths during and after 5-min forearm arterial occlusion. The measurements were performed in 14 healthy young adults using a two-channel, two-separation bbNIRS probe holder strapped on the brachioradialis muscle below the cuff proximal to the wrist, with a sampling rate of 0.67 Hz. After data processing and group averaging, we obtained several key findings. (1) During the 5-min forearm arterial occlusion, Δ[oxCCO] remained constant, as observed in both the 1- and 3-cm channels, and this observation was consistent with two previous reports. (2) A linear correlation between Δ[HbO] and Δ[oxCCO] was obtained only during the recovery phase, with a stronger correlation in deeper tissues than in shallower tissues. (3) The constant course of Δ[oxCCO] during the arterial occlusion period can be explained by the assumption that skeletal muscle oxygen metabolism during arterial occlusion remains unchanged before all oxy-hemoglobin (and oxy-myoglobin) resources are completely depleted. Because a 5-min arterial occlusion is not adequate to exhaust all oxygen supply in the vascular bed of the forearm during arterial occlusion, the local oxygen supply to cellular mitochondria maintains redox metabolism uninterrupted by occlusion. Overall, this study offers an excellent example of how bbNIRS can be used to non-invasively investigate mitochondrial metabolism and/or activity in response to different physiological conditions.

## 6 Appendix A: A Data Processing Flow Chart Used to Quantify Δ[HbO], Δ[HHb], and Δ[oxCCO] from Raw bbNIRS Data

**Fig. 6:**
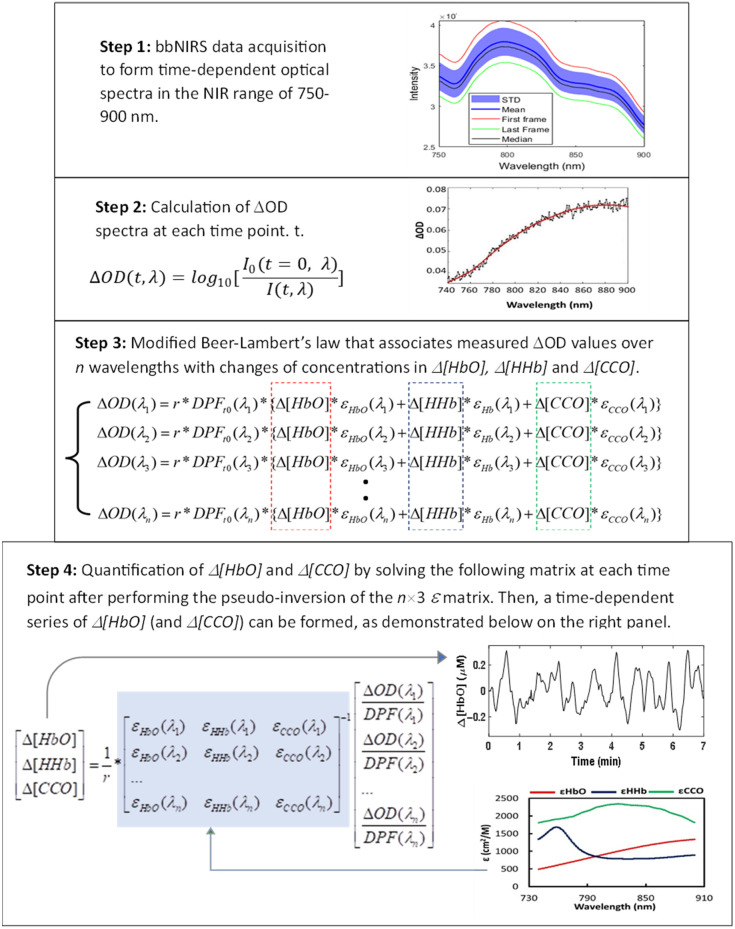
A data processing flow chart used to quantify Δ[HbO], Δ[HHb], and Δ[CCO] from raw bbNIRS data.

Figure 6 shows graphically four steps, as described in detail in Sec. 2.3. These steps were used to derive changes in [HbO], [HHb], and [oxCCO] from raw bbNIRS measurements during the entire arm-cuff experiment. Note that the notation of [CCO] in Steps 3 and 4 represent [oxCCO].

## 7 Appendix B: Correlations between 1- and 3-cm Separations for Δ[HbO], Δ[HHb], and Δ[oxCCO]

**Fig. 7:**
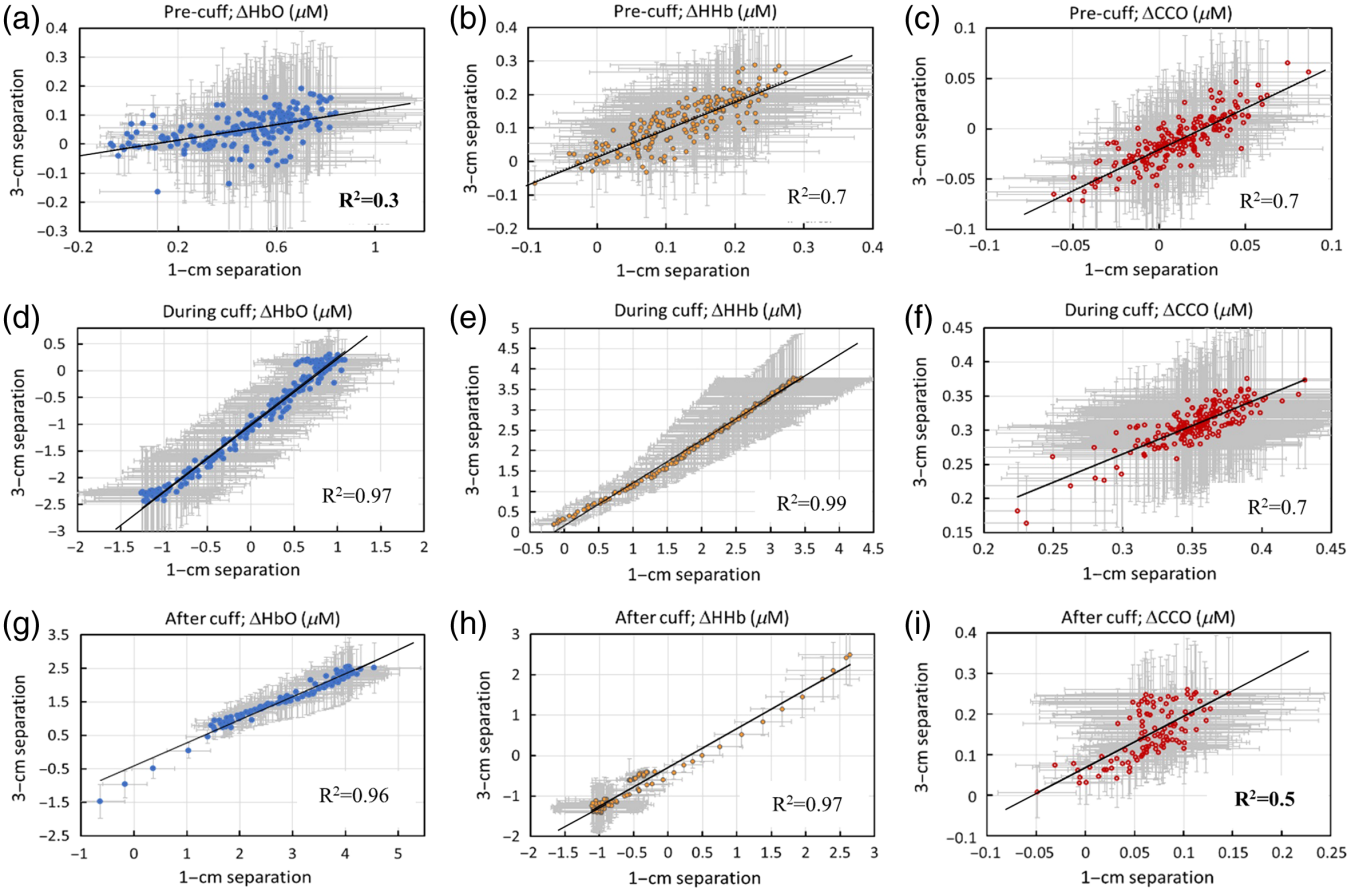
Correlations between 1- and 3-cm separations during (a)–(c) baseline (or pre-cuff), (d)–(f) arterial occlusion, and (g)–(i) recovery for Δ[HbO], Δ[HHb], and Δ[oxCCO]. The error bars represent the standard error of the mean for respective concentrations.

Figure 7 shows the dynamic relationships of each chromophore taken with 1-cm and 3-cm channels during each of the three dynamic phases, namely, pre-cuff, during-cuff, and post-cuff periods. The correlations between the two channels from two tissue depths were high ($R^{2}>0.7$) in most cases (7 out of 9 cases), except two situations: (i) during the baseline for Δ[HbO] [Panel (a); $R^{2}=0.3$] and (ii) during the recovery phase for Δ[oxCCO] [Panel (i); $R^{2}=0.5$]. These two special cases are marked in Figs. 4(a) and 4(c) by purple arrows, respectively.

## Data Availability

The data presented in this study are available on request from the corresponding author.
